# Immune Reconstitution after Haploidentical Hematopoietic Stem Cell Transplantation with Different Non‐T‐Cell Depletion Protocols

**DOI:** 10.1002/mco2.70206

**Published:** 2025-05-19

**Authors:** Xiao‐Di Ma, Zheng‐Li Xu, Xiao‐Jun Huang

**Affiliations:** ^1^ Peking University People's Hospital, Peking University Institute of Hematology, National Clinical Research Center for Hematologic Disease, Beijing Key Laboratory of Hematopoietic Stem Cell Transplantation Peking University Beijing China; ^2^ Peking‐Tsinghua Center for Life Sciences, Academy for Advanced Interdisciplinary Studies Peking University Beijing China; ^3^ State Key Laboratory of Natural and Biomimetic Drugs Peking University Beijing China

**Keywords:** immune reconstitution, haploidentical hematopoietic stem cell transplantation, conditioning regimen, posttransplant cyclophosphamide

## Abstract

Haploidentical hematopoietic stem cell transplantation (haplo‐HSCT) has emerged as a critical treatment for hematological diseases. However, challenges, such as graft rejection and graft‐versus‐host disease (GVHD), have historically been faced with this procedure. Immune reconstitution (IR) has been shown to have profound effects on posttransplantation complications, such as relapse, infections, and GVHD. Recent advances in non‐T‐cell depletion protocols including the Beijing protocol and Baltimore protocol have significantly influenced the outcomes of haplo‐HSCT by improving IR. Clinical studies and multiomic analyses have revealed that different protocols offer distinct mechanisms for IR patterns and further influence clinical outcomes. However, there is a lack of comprehensive reviews that systematically link the differences in IR between two protocols to their clinical outcomes, which leaves a critical gap in understanding the optimal strategies for IR in haplo‐HSCT. This review provides an analysis of IR following haplo‐HSCT with different protocols; it compares the clinical outcomes of various protocols, addresses the role of each immune cell subset in influencing outcomes and discusses emerging strategies aimed at improving IR. This review highlights the importance of ongoing research for improving immune reconstitution strategies, ultimately reducing posttransplant complications and offering targeted treatments to improve patient outcomes.

## Graphical abstract

This review examines immune reconstitution dynamics after haploidentical hematopoietic stem cell transplantation using different non‐T‐cell depletion protocols, including the Beijing and Baltimore protocols. It summarizes the reconstitution patterns of immune cell subsets and their correlation with posttransplant complications like relapse, GVHD, and survival. Emerging strategies to enhance immune reconstitution are also discussed, aiming to improve clinical outcomes.

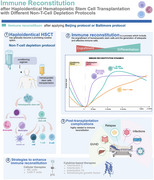

## 1 Introduction

Haploidentical hematopoietic stem cell transplantation (haplo‐HSCT) has gradually become a promising curative option for a variety of hematological indications and is one of the most widely used alternative donor techniques [1, 2, 3, 4]. The success of HSCT depends on the engraftment of hematopoietic stem cells and the generation of adequate and effective immune cells, a process known as immune reconstitution (IR) [5, 6, 7, 8]. IR has been shown to have profound effects on posttransplantation complications such as relapse, infections, and graft‐versus‐host disease (GVHD), highlighting its crucial role in HSCT [9, 10, 11, 12, 13]. Numerous factors can impact the efficacy of IR, including the conditioning regimen, donor age and sex, histocompatibility, the stem cell source, and the GVHD prophylaxis strategy [5, 14, 15].

To improve the outcomes of haplo‐HSCT, some centers have adopted T‐cell depletion (TCD) strategies, which help reduce the rate of GVHD [16, 17]. However, these transplants tend to have relatively high rates of graft rejection, slow IR, and a substantially high risk of treatment‐related mortality [16, 18, 19]. In recent decades, unmanipulated haploidentical transplantation, which is performed without ex vivo TCD, has emerged as a viable option, with its frequency and success steadily increasing [20, 21]. Among the various strategies, the granulocyte colony‐stimulating factor (G‐CSF)/antithymocyte globulin (ATG)‐based Beijing protocol and the posttransplantation cyclophosphamide (PTCy)‐based Baltimore protocol have proven to be the most effective prophylactic approaches [22, 23]. Many studies have indicated that these two different strategies have different effects on IR and distinct clinical outcomes, owing to their different underlying molecular mechanisms [24, 25, 26, 27, 28]. Recent high‐dimensional cytometry and multiomic studies have provided a more in‐depth view of immune system dynamics after transplantation [29, 30, 31].

Immune recovery occurs in distinct phases, with innate immunity, including monocytes, granulocytes, dendritic cells (DCs), and natural killer (NK) cells, recovering within weeks to months, whereas adaptive immunity follows a slower trajectory [32]. B‐cell and CD8^+^ T‐cell recovery is typically observed within 100 days to 6 months post‐HSCT [28, 33], whereas thymic‐dependent CD4^+^ T‐cell reconstitution may take up to 9 months [27]. Initial T‐cell expansion primarily involves CD8^+^ memory T cells, which respond to preexisting antigens but have a limited capacity for novel immune challenges. Full T‐cell reconstitution requires thymic differentiation of lymphoid progenitors into naive T cells [34, 35]. The kinetics of immune recovery are closely linked to posttransplant complications, including infections, graft failure, and GVHD, highlighting the critical role of IR in transplant success [36–10, 38].

Here, we provide a brief overview of two main non‐TCD strategies for haplo‐HSCT and present a comprehensive review of the IR dynamics of individual immune cell subsets, including innate and adoptive immune cells, following haplo‐HSCT with the Beijing and Baltimore protocols. We thoroughly examine the associations between posttransplantation complications and the recovery of specific immune cell subsets. Furthermore, we explore recent advances aimed at enhancing immune recovery after transplantation.

## 2 The Evolution of Haplo‐HSCT and Non‐TCD Strategies

Most patients who need HSCT lack suitable HLA‐matched donors, necessitating the use of alternative donors and the development of haplo‐HSCT. Moreover, the easier accessibility of haploidentical donors allows for quick administration of stem cell boosts, cellular therapies using antileukemic effector cells or antigen‐specific T cells, and even the production of a second stem cell graft in a short time. In contrast, transplantation from a fully haplotype‐mismatched donor leads to significant bidirectional alloreactivity, including both graft‐versus‐host and host‐versus‐graft reactions. Therefore, the wider application of haplo‐HSCT has been limited by a high incidences of graft rejection and GVHD caused by the HLA mismatch. To address these challenges, several strategies have been developed.

**FIGURE 1:**
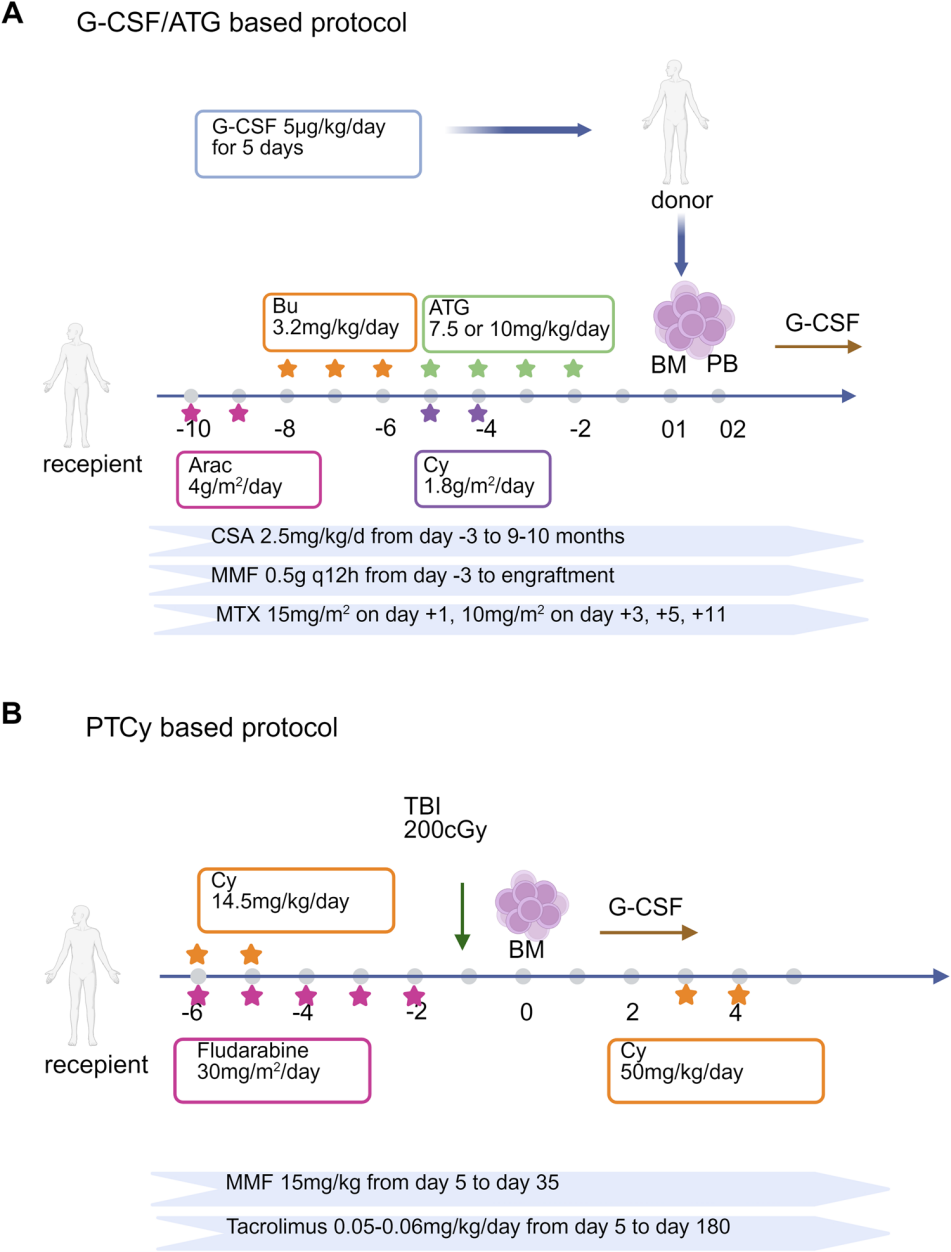
The standard conditioning regimens with (A) the granulocyte colony‐stimulating factor (G‐CSF)/antithymocyte globulin (ATG)‐based Beijing protocol and (B) the posttransplantation cyclophosphamide (PTCy)‐based Baltimore protocol. (Figure was created with Biorender.com).

Ex vivo TCD by CD34^+^ selection in haploidentical grafts was the original standard method in the early days, which could reduce the risk of GVHD but delay the immune recovery and lead to increased graft failure and infections [39, 40]. Techniques for ex vivo TCD have undergone a constant evolution and have shifted from a broad TCD methodology to more selective TCD approaches [41, 42, 43, 44, 45]. These techniques have advanced, with the next generation replacing positive selection of hematopoietic progenitor cells by depleting CD3^+^ T cells, thereby increasing the number of accessory cells, such as monocytes, DCs, and NK cells in the graft [42, 46]. Alternatively, selective depletion of αβ^+^ T lymphocytes preserves NK cells and γδ T cells in the graft [44, 47]. However, these advanced methods are often more costly and require highly experienced centers. Therefore, in recent years, unmanipulated haploidentical transplantation without ex vivo TCD has become a promising option, with increases in frequency and success rate. Among various non‐TCD strategies, the use of ATG and PTCy has proven to be the most effective prophylactic approach [48] (Figure 1). Several non‐randomized studies have shown that survival outcomes after haploidentical HSCT using the G‐CSF/ATG‐based protocol and PTCy‐based protocol are comparable to those of matched sibling donor transplantation (MSDT) or matched unrelated donor (MUD) transplantation [49, 50, 51]. Table 1 summarizes the reported clinical outcomes of patients receiving haplo‐HSCT with the two protocols from recent studies over the past 5 years.

**TABLE 1:** Reported outcomes of patients who received haplo‐SCT with a G‐CSF/ATG‐based protocol or PTCy‐based protocol.

					GVHD					
Study	Disease	*N*	Doses of ATG or PTCy	Graft source	Grade II–IV/III–IV aGVHD	cGVHD	Engraftment rates	Relapse	OS	FFS
G‐CSF/ATG‐based protocol										
Wang, 2019 [52]	Hematological malignances	125	ATG 10 mg/kg	G‐primed BM+PB	36%/18%	44%/16% at 2‐y	100%	14% at 2‐y	77% at 2‐y	71% at 2‐y
Tang, 2020 [53]	AML/ALL/MDS/CML/NHL	176	ATG 10 mg/kg	G‐primed BM+PB	26.7%/8%	42.3%/9.5% at 3‐y	96.6%	14.9% at 3‐y	78.3% at 3‐y	74.3% at 3‐y
Lu, 2021 [54]	AML	377	ATG 7.5‐10 mg/kg	G‐primed BM+PB	30.4%/13.7%	56.4%/19.7% at 3‐y	98%	14.3% at 3‐y	74.9% at 3‐y	73.8% at 3‐y
Tsai, 2022 [26]	AML/ALL/MDS/MPN/CML/NHL/HL/myeloma	110	ATG 6 mg/kg	G‐primed BM+PB	NA/16.4%	38.8%/NA at 2‐y	99.3%	34.5% at 1‐y	48.9% at 2‐y	NA
Xu, 2022 [55]	SAA	183	ATG 10 mg/kg	G‐primed BM+PB	31.3%/8.9%	29.3/7.0% at 5‐y	97.3%	NA	87.1% at 9‐y	86.5% at 9‐y
Barkhordar, 2022 [56]	AML/ALL	40	ATG 10 mg/kg	G‐primed PB	57.5%/30.0%	38.23%/NA at 1‐y	95%	17.5 at 5‐y	47.5% at 5‐y	42.5% at 5‐y
Yao, 2022 [57]	SAA	35	ATG 10 mg/kg	G‐primed BM+PB	42.9%/NA	51.3%/NA at 3‐y	100%	NA	92.8% at 3‐y	90.4% at 3‐y
Zhang, 2023 [58]	AML/ALL/MDS/lymphoma	61	ATG 10 mg/kg	G‐primed BM+PB	39.3%/24.6%	39.9%/19.4% at 2‐y	100%	16.2% at 2‐y	55.0% at 2‐y	54.1% at 2‐y
Cao, 2023 [59]	AML/MDS	40	ATG 10 mg/kg	G‐primed BM+PB	32.8%/12.5%	27.2%/19.6% at1‐y	100%	30.4% at 2‐y	57.3% at 2‐y	34.8% at 2‐y
Xu, 2024 [60]	AML/ALL/MDS	230	ATG 10 mg/kg	G‐primed BM+PB	28.6%/10.8%	33.4%/10.6% at 3‐y	98.3%	14.3% at 3‐y	84.1% at 3‐y	77.8% at 3‐y
Xiao al, 2025 [61]	HLH	42	ATG 7–9 mg/kg	G‐primed BM+PB	28.6%/26.2%	57.2%/NA at 2‐y	100%	NA	78.4% at 2‐y	71.3% at 2‐y
PTCy‐based protocol										
Makanga, 2020 [62]	Hematological malignancies	32	PTCy 100 mg/kg	PB	59%/19%	21% at 2‐y	100%	38% at 2‐y	59% at 2‐y	47% at 2‐y
Sugita, 2021 [63]	AML/ALL/MDS/lymphoma	82	PTCy 100 mg/kg	G‐primed PB	24%/1%	28%/15% at 2‐y	96%	37% at 2‐y	58% at 2‐y	48% at 2‐y
Saglio, 2021 [64]	AML/ALL	23	PTCy 100 mg/kg	BM or PB	8%/NA	5%/NA	73%	33% at 5‐y	75% at 5‐y	NA
Battipaglia, 2022 [65]	AML	374	PTCy 100 mg/kg	PB	34%/12%	33%/10% at 2‐y	97%	25% at 2‐y	58% at 2‐y	53% at 2‐y
Baron, 2022 [66]	AML(NR)	762	NA	NA	23%/8%	21%/7% at 2‐y	NA	50% at 2‐y	28% at 2‐y	26% at 2‐y
Dezern, 2023 [67]	SAA	27	PTCy 100 mg/kg	BM	7%/NA	4%/NA at 1‐y	88.9%	NA	92% at 1‐y	NA
Dulery, 2023 [68]	AML/ALL/CLL/MM/MDS/MPN/lymphoma	38	PTCy 100 mg/kg	PB	33%/13%	35%/16% at 2‐y	100%	20% at 2‐y	56% at 2‐y	49% at 2‐y
		55	PTCy 80 mg/kg		32%/16%	41%/13% at 2‐y	100%	19% at 2‐y	70% at 2‐y	65% at 2‐y
Arslan, 2024 [69]	AML/ALL/MDS/CML/CLL	104	PTCy 100 mg/kg	PB	40%.7%	52.9%/22.1% at 2‐y	100%	15% at 2‐y	78% at 2‐y	73% at 2‐y
Ruggeri, 2024 [70]	AML	96	NA	BM or PB	36.7%/14.4%	22.4%/6.6% at 2‐y	90.4%	19.5% at 2‐y	71.5% at 2‐y	69.5% at 2‐y
Harada, 2025 [71]	Hematological diseases	1142	NA	NA	26.4%/7.0%	18.6%/9.4% at 2‐y	93.7%	34.2% at 2‐y	56.2% at 2‐y	NA

Abbreviations: aGVHD: acute graft‐versus‐host disease; ALL: acute lymphoblastic leukemia; AML: acute myeloid leukemia; BM: bone marrow; cGVHD: chronic GVHD; CML: chronic myeloid leukemia; FFS: failure‐free survival; HL: Hodgkin lymphoma; MDS: myelodysplasia; MPN: myeloproliferative neoplasm; NA: not applicable; NHL: non‐Hodgkin lymphoma; NR: not remission; OS: overall survival; PB: peripheral blood; SAA: severe aplastic anemia.

### 2.1 The G‐CSF/ATG‐Based Protocol (Beijing Protocol)

Huang et al. [72] from Peking University established the G‐CSF/ATG‐based protocol, which includes unmanipulated G/CSF‐primed bone marrow (BM) and peripheral blood (PB) grafts; administration of ATG to recipients; and cyclosporine, mycophenolate mofetil, methotrexate for GVHD prophylaxis [20, 73]. ATG exhibits a relatively prolonged half‐life in vivo, remaining detectable for more than 30 days postadministration. This extended presence enables sustained depletion of T cells, effectively preventing GVHD without increasing the rate of relapse. Moreover, the inclusion of ATG in the regimen facilitates faster donor chimerism following HSCT from haplo‐donors. G‐CSF has been identified as a novel mediator that induces T‐cell tolerance. It makes this effect by shifting T cells from the Th1 phenotype to the Th2 phenotype, producing a balance favoring T regulatory cells over Th17 cells, and manipulating the activities of DCs and myeloid‐derived suppressor cells.

The initial result of a cohort study involving 58 patients with leukemia treated with the Beijing protocol was published in 2004; all patients achieved successful engraftment, although 37.9% experienced Grade II–IV acute GVHD (aGVHD) and 5.2% developed Grade III–IV aGVHD. The estimated 2‐year disease‐free survival (DFS) for the cohort was 67.2%. In recent decades, this protocol has been developed to include personalized conditioning and graft modifications, non‐HLA‐based donor selection, and risk stratification‐based interventions to reduce the risk of GVHD and relapse [74, 75, 76, 77]. Recently, Wang et al. [78] reported the long‐term outcomes of a cohort of 756 patients who underwent haplo‐HSCT using the Beijing protocol, with 99% achieving full donor chimerism. The 2‐year relapse rates were 15% for standard‐risk patients and 26% for high‐risk patients. Among the cohort, 480 patients survived, with 3‐year DFS rates of 68% for the standard‐risk group and 49% for the high‐risk group [78]. In terms of nonmalignant diseases, multicenter studies conducted by Xu et al. [79] also demonstrated promising outcomes; during long‐term follow‐up, 97.5% of the 275 evaluable patients with severe aplastic anemia achieved sustained full donor chimerism, and 93.4% achieved complete hematopoietic recovery. At 9 years, the estimated overall survival (OS) rate was 85.4 ± 2.1%, and the failure‐free survival rate was 84.0 ± 2.2% [79]. These findings indicate an expansion of disease indications, enabling successful haploidentical transplants for treating not only hematological malignancies but also nonmalignant conditions. Moreover, Huang and his colleagues [80] recently modified the graft source and reported that the comparable clinical outcomes between the G‐CSF primed PB (G‐PB) group and the G‐BM plus G‐PB groups in patients with hematologic malignancies.

### 2.2 The PTCy‐Based Protocol (Baltimore Protocol)

Another recognized approach was designed by hematologists from Baltimore, who administered high‐dose PTCy with a combination of fludarabine, low‐dose Cy and a total body irradiation regimen for 2 days. Cyclophosphamide‐induced allogeneic tolerance is believed to be primarily achieved through the selective elimination and suppression of proliferating alloreactive T cells. On days +3 and +4 post‐HSCT, alloreactive T cells are in their peak phase of proliferation, which makes them especially susceptible to cyclophosphamide‐induced cytotoxicity. In contrast, other resting memory cells and regulatory T cells (Treg cells) are relatively resistant to Cy, which allows them to survive and provide immune protection against infections until a new T‐cell repertoire is established. Additionally, the stem cell component of the graft is highly resistant to Cy, as aldehyde dehydrogenase activity helps prevent the drug from entering the stem cells. This specificity makes Cy‐induced killing an ideal method for inducing tolerance in allogeneic transplants by targeting both donor‐derived and recipient‐derived alloreactive T cells. The cardiotoxicity of PTCy has been reported at a total dose of 100 mg/kg [81], with clinical consequences including atrial fibrillation, heart failure, and death. It has been reported that reducing the dosage to 40 mg/kg × 2 or 25 mg/kg × 2 remains effective [63, 82]. Some recent clinical trials have begun to assess the clinical prognosis of patients receiving reduced‐dose PTCy [83, 84].

A study involving 1,939 patients with acute myeloid leukemia (AML) who underwent haploidentical HSCT using the PTCy protocol, conducted by the European Society for Blood and Marrow Transplantation (EBMT), reported a graft failure rate of 6% and 2‐year OS rate was 60.9% (95% confidence interval [CI], 58.4%–63.3%) [85]. Raj et al. [86] retrospectively analyzed 55 patients who underwent haplo‐HSCT with PTCy, and 96% of the patients achieved successful engraftment. The incidence of Grade II acute GVHD was 51%, while that of Grade III–IV acute GVHD was 8%, and the OS at 2 years was 48% [86].

The Center of International Blood and Marrow Transplant Research (CIBMTR) conducted a comparative study of younger haploidentical donors (≤35 years) with the use of the PTCy protocol and older MUDs (>35 years) with the use of conventional GVHD prophylaxis in two recipient patient cohorts with AML/MDS. The results showed that the younger haploidentical donors treated with PTCy could achieve better outcomes than the older MUDs using standard prophylaxis. Compared with those receiving transplants from older MUDs, recipients of transplants from younger haploidentical donors presented superior 4‐year OS (hazard ratio [HR] = 0.81, *p* = 0.01), a lower cumulative incidence of 4‐year nonrelapse mortality (NRM) (HR = 0.59, *p* = 0.02), a reduced incidence of 100‐day Grade II–IV aGVHD (HR = 0.64, *p* < 0.001) and a lower incidence of 2‐year chronic GVHD (cGVHD) (HR = 0.49, *p* < 0.001) than those receiving transplants from older MUDs [87]. In the ALL cohort, outcomes with younger haploidentical donors (*N* = 187) were compared with outcomes with older MUD donors (*N* = 232). Compared with transplantation from younger haploidentical donors, transplantation from older MUDs was associated with a greater incidence of 2‐year cGVHD (HR = 1.91, *p* = 0.002), increased 4‐year NRM (HR = 2.75, *p* = 0.001), and inferior 4‐year OS (HR = 1.77, *p* = 0.08) [88].

In summary, both protocols aim to achieve the same result through different mechanisms: establishing an appropriate IR pattern to balance graft failure, GVHD, and infections as effectively as possible.

## 3 The Patterns of IR with Different Protocols

**FIGURE 2:**
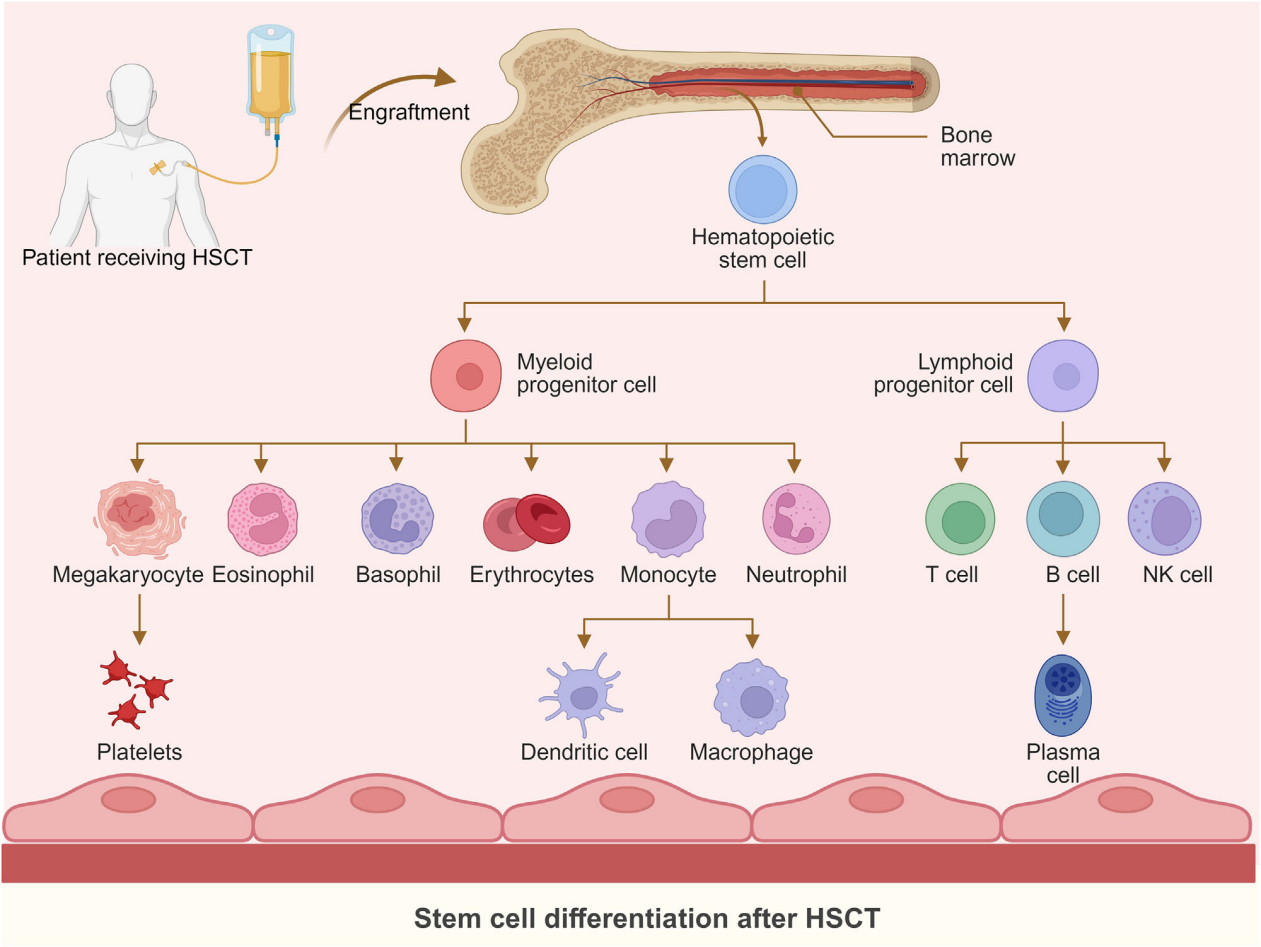
Overview of immune cell differentiation after HSCT. The figure illustrates the various immune cell types and their development from distinct progenitors. Monocytes, granulocytes, and dendritic cells originate from myelomonocytic progenitor cells, while natural killer (NK) cells, B cells, and T cells arise from lymphoid progenitors. These cells require specific microenvironments for efficient differentiation from primitive progenitors, often leading to delayed and incomplete recovery. (Figure was created with Biorender.com).

Post‐HSCT immune recovery is a complex and dynamic process involving the reconstitution of various cell subsets (Figure 2). This process unfolds in distinct phases, with innate immunity recovering first, followed by adaptive immunity.

Guo et al. [29] recently demonstrated distinct patterns of immune homeostasis remodeling between haplo‐HSCT and MSDT. Studies utilizing multiomic techniques, including single‐cell RNA sequencing, have revealed that although all immune subpopulations from donors are restored in recipients, their relative abundances vary. Notably, in haplo‐HSCT, unique T‐cell clonotypes, particularly those expressing thymus development‐related genes, emerge in recipients [29]. These findings suggest that thymic regeneration plays an essential role in reconstituting the immune system via the central pathway, which is important for long‐term immune tolerance. In contrast, MSDT recipients exhibit a predominance of consistent T‐cell clonotypes, indicating a reliance on peripheral reconstitution. These findings emphasize the importance of thymus‐dependent central T‐cell regeneration in haplo‐HSCT and suggest that strategies aimed at promoting thymic function could improve IR and clinical outcomes. Some studies have employed advanced techniques such as multiomic techniques and T‐cell repertoire analysis to elucidate the dynamics of immune system recovery posttransplantation [89, 90]. These analyses help to identify rare immune cell subsets and biomarkers associated with effective IR or a heightened risk of posttransplantation complications [30].

Numerous factors influence IR, and emerging evidence suggests that the patterns of immune recovery after haplo‐HSCT differ between the G‐CSF/ATG‐based protocol and PTCy‐based protocol, each exhibiting unique characteristics (Table 2). G‐CSF plays a crucial role in inducing T‐cell tolerance [91]. ATG is widely recognized for its ability to delay the recovery of CD3^+^ and CD4^+^ T cells, especially Th cells, whereas NK cells are maintained at high frequencies and numbers during the first month [92, 93]. PTCy targets proliferating cells, including NK and CD8^+^ T cells, but preserves significant numbers of CD4^+^ T cells and Treg cells, facilitating their reconstitution [94]. Several studies have indicated that PTCy selectively eliminates alloreactive T cells through direct destruction during proliferation and intrathymic clonal deletion of precursors, leading to bidirectional T‐cell tolerance [95]. Following the application of two different conditioning regimens, innate and adaptive immunity sequentially reconstituted; however, the kinetics of immune cell recovery at each stage varied across different protocols.

**TABLE 2:** Key points and recovery dynamics of each immune cell subset after applying haplo‐SCT with a G‐CSF/ATG‐based protocol or PTCy‐based protocol.

Immune cell subsets		Key points	G‐CSF/ATG‐based haplo‐SCT protocol	PTCy‐based haplo‐SCT protocol	Relationship with prognosis
Innate immune cells	Monocytes	‐ Release chemokines and cytokines; ‐ Contribute to the innate immune response and initiate acquired immunity.	‐ Recovered rapidly and reached normal counts at days +15–30 post‐HSCT [24, 96]; ‐ Median counts of 567.84 cells/µL at day +30 posttransplantation, compared with those who underwent MSDT [96].	‐ Recovered to 100–400 cells/µL as soon as day +30 [97].	‐ The total monocyte level >0.3 × 10^9^ cells/L at day +100 was statistically significantly associated with better survival outcomes [98].
	Neutrophils	‐ The first cell group to recover; ‐ Serve for validation of successful engraftment.	‐ Took a median time of 13 days (range 9–25 days) to achieve neutrophil recovery [49].	‐ The median times were 16 days for the PB graft source and 17 days for the BM graft source [99].	‐ Deficient neutrophil maturation diminished macrophage‐induced inflammation [100].
	Natural killer cells	‐ The first reconstituted lymphocyte cells; ‐ Important for immune tolerance and surveillance.	‐ The recovery of NK cells was rapid, with a fast increase in the CD56^bright^ NK subset and a corresponding reduction in the CD56^dim^ NK subset during the first 2 month after transplantation [101].	‐ Reached 19 and 51 cells/µL at days +30 and +100 post‐HSCT, respectively [102].	‐ Quicker recovery of CD56^bright^ NK cells was connected to lower rates of relapse [101]. ‐ KIR mismatching was associated with a worse survival because of an increased rate of acute GVHD [103].
	γδ T cells	‐ Usually reside in the mucosa and skin; ‐ Play a significant role in both innate and adaptive immunity.	‐ Recovery was continuously delayed for at least 180 days after HSCT [104].	‐ The γδ T‐cell counts remained significantly low at day +30 posttransplantation [105].	‐ High γδT‐cell counts post‐HSCT were associated with a lower incidence of infection [106]; ‐ Impaired γ/δ T‐cell recovery was associated with an increased load of the Epstein–Barr virus load [105].
Adaptive immune cells	CD4^+^ T cells	‐ Markedly slow recovery after HSCT, usually being the last subset to recover.	‐ Reached median counts of 24.31, 93.12, 109.71, 173.43, and 305.86 cells/µL at days +30, +60, +90, +180, and +365 posttransplantation, respectively [96].	‐ Reached 64 and 149 cells/µL at days +40, +100 after HSCT, respectively [102].	‐ The counts of CD4^+^ T cells at days +30 and +90 were not associated with the survival (Beijing protocol).
	CD8^+^ T cells	‐ Recover earlier than CD4^+^ T cells after haplo‐HSCT, leading to an inverted ratio of CD4^+^/CD8^+^ cells.	‐ Reached median counts of 71.92, 493.93, 684.00, 945.63, and 1035.30 cells/µL at days +30, +60, +90, +180 and +365 posttransplantation, respectively [96].	‐ Reached 148 and 107 cells/µL at days +40, +100 after HSCT, respectively; achieved a normal level by day +180 posttransplantation [102, 107].	‐ The CD8^+^ T‐cell level ≥375 cells/µL at day +90 was correlated with decreased bacterial infection and NRM, improved LFS and OS [28].
	Treg cells	‐ Crucial mediators in promoting immune tolerance and preventing GVHD	‐ Reached median counts of 11.18, 19.07, 23.29, 48.58, 91.28 cells/µL at days +30, +60, +90, +180 and +365 posttransplantation, respectively [96].	‐ Recovered quickly and exhibited at high levels in patients with GVHD [94]; ‐ Achieved normal levels from day +30 [107]	‐ The expression of [108] ALDH in Tregs, which confers resistance to Cy, may play a role in the clinical efficacy of PTCy in preventing GVHD [94].
	B cells	‐ It takes 6–12 months for the absolute count to reach a normal level; ‐ Functional reconstitution takes several years.	‐ Reached median counts of 5.28, 11.00, 11.26, 69.84, 242.95 cells/µL at days +30, +60, +90, +180, and +365 posttransplantation, respectively [96].	‐ B cells were not detected up to 4 weeks and appeared at week +5; the levels increased from week +7, reaching 50–500 cells/µL [109]; ‐ B‐cell recovery after 4 weeks was prominent in PTCy‐based haplo‐HSCT [108].	‐ A high proportion of transitional B cells was significantly associated with a low incidence of chronic GVHD [108].

Abbreviations: ALDH: aldehyde dehydrogenase; LFS: leukemia‐free survival; MSDT: matched sibling donor transplantation; NRM: nonrelapse mortality; OS: overall survival.

### 3.1 The Reconstitution of Innate Immune Cells

**FIGURE 3:**
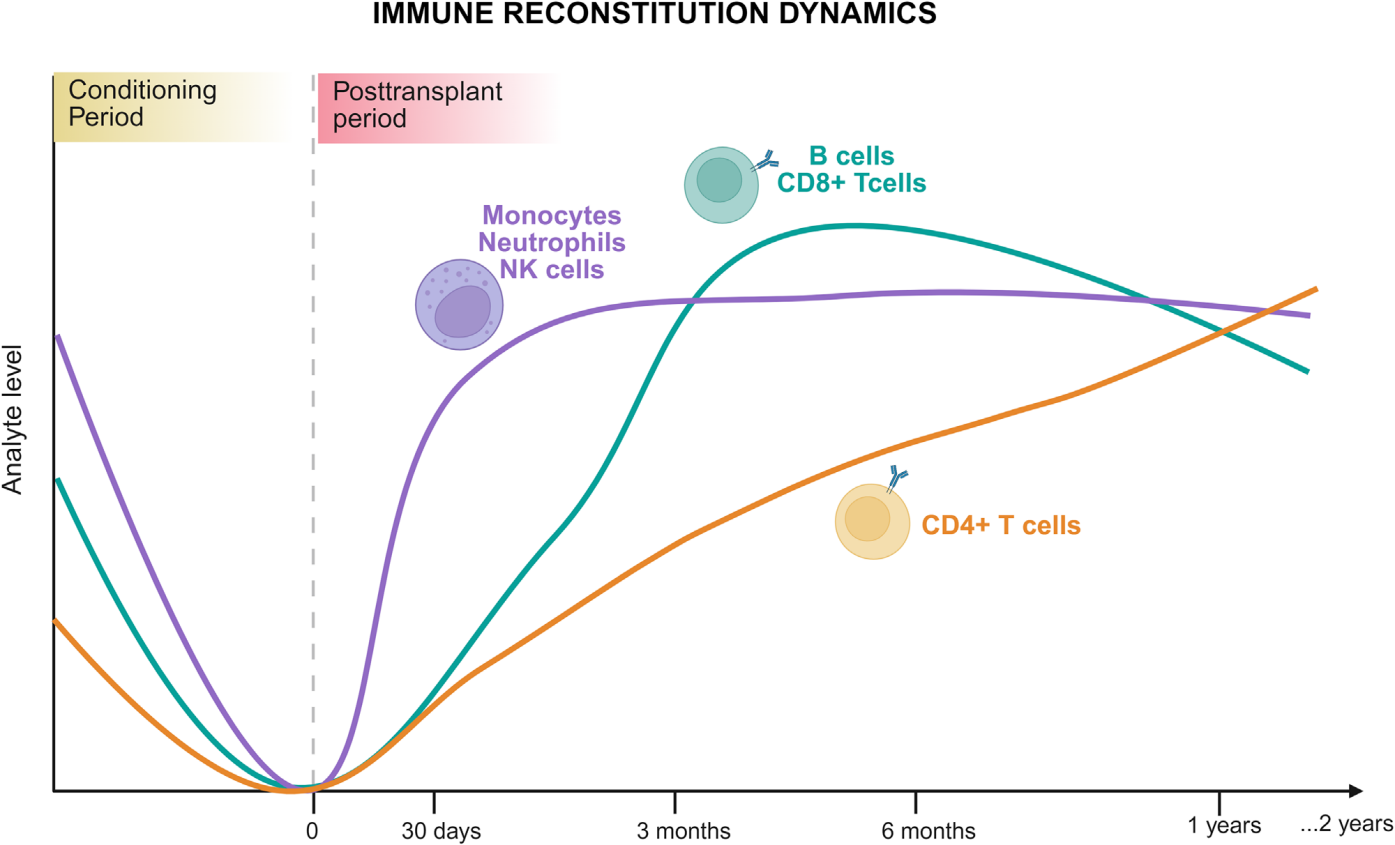
Dynamics of the immune reconstitution following haplo‐HSCT. The graph illustrates the immune recovery of key cell subsets, including monocytes, neutrophils, and NK cells (purple); CD4+ T cells (orange); and CD8+ T cells and B cells (green) over time. The *x*‐axis represents the posttransplantation period (from conditioning to 2 years posttransplantation), and the *y*‐axis represents the analyte levels (immune cell counts). The conditioning period and posttransplantation period are indicated on the graph. Adapted with permission from Ref. [34], Springer Nature. (Figure was created with Biorender.com).

Innate immune cells, including monocytes, granulocytes, DCs, and NK cells, typically recover within weeks to months after HSCT [110] (Figure 3). Several studies have indicated that the reconstitution of innate immune cells is significantly faster than that of adaptive immune cells, which are crucial for immunological defense [111].

#### 3.1.1 Monocytes

Monocytes, which originate from the BM, circulate into the PB and differentiate into tissue macrophages. Monocytes exhibit heterogeneity and can be categorized into three subtypes: classic (CD14^++^CD16^−^), intermediate (CD14^++^CD16^+^), and nonclassic (CD14^+^CD16^++^) monocytes. Their key roles include phagocytosis and the release of both proinflammatory/anti‐inflammatory chemokines and cytokines, which contribute to the innate immune response and the initiation of acquired immunity. In patients who underwent haplo‐HSCT with the Beijing protocol, monocytes recovered rapidly and reached normal counts at 30 days after transplantation, which was comparable to patients who underwent HLA‐identical sibling HSCT [25]. Similarly, Chang et al. [24] reported that monocytes recovered promptly to normal counts by day +15 after haplo‐HSCT with the Beijing protocol. Univariate analysis revealed no associations between recipient age, sex, or underlying disease and monocyte recovery. Retière et al. [97] reported that, compared with the PTCy regimen, the ATG regimen was associated with a higher median monocyte count on day +30 after transplantation. A few studies have demonstrated a relationship between monocyte recovery and better outcomes after allogeneic HSCT [98]. However, there are limited data on the IR of monocytes following haplo‐HSCT with PTCy regimens. Further research is needed to explore the patterns of monocyte reconstitution, the relationships of these patterns with outcomes, and the underlying mechanisms involved.

#### 3.1.2 Neutrophils

Neutrophils constitute the first immune cell group to recover following HSCT. Neutrophil recovery serves as a validation of successful engraftment, which is said to occur when the absolute neutrophil count reaches 0.5 × 10^9^/L for 3 consecutive days. However, despite the rapid recovery of neutrophil counts, their functions—including chemotaxis, phagocytosis, superoxide production, and bacterial killing—remain suboptimal during the immediate posttransplant period and gradually normalize within 2 months [112]. Massoud et al. [31] reported that neutrophil engraftment was notably delayed in a PTCy group, occurring at a median of 12 days, compared with 10 days in a group that received ATG. Tsai et al. [26] compared the engraftment kinetics between a Beijing protocol group and a PTCy group and reported that the Beijing protocol group had a more favorable neutrophil engraftment time (median 12 days) than did patients receiving PTCy (median 32 days). Wang et al. [76] conducted a study in which the Beijing protocol was used, and the median time to reach neutrophil engraftment was 13 days for patients with malignant diseases. In a report on PTCy protocol use, the cumulative incidence of engraftment on day +30 was 89%, with a median time of 19 days [85]. Neutrophils possess strong phagocytic capabilities akin to those of macrophages, and neutrophils are equipped with a distinctive set of microbicidal agents that are swiftly deployed upon encountering pathogens. Moreover, recent evidence has suggested that neutrophils also influence adaptive immunity. Neutrophils can act indirectly through antigen‐presenting cells (APCs) or directly through T cells. Activated neutrophils modulate DC maturation and trafficking, whereas infected neutrophils can serve as substrates for antigen cross‐presentation by DCs.

#### 3.1.3 Dendritic Cells

DCs are important APCs that display antigen peptides on major histocompatibility complex (MHC)‐I and MHC‐II molecules on their surfaces. Following antigen uptake, DCs migrate to lymph nodes to activate T cells and B cells, leading to an adaptive immune response. Porta et al. [113] conducted a study to explore the kinetics of the reconstitution of two circulating DC subsets. The first subset was myeloid DCs (DC1s), which activate Th1 responses, leading to the secretion of interferon (IFN)‐γ and the generation of cytotoxic lymphocytes and environmental antigens; the other subset is plasmacytoid DCs (DC2s), which are strong producers of the antiviral cytokine IFN‐α, which is crucial for controlling viral infections. Researchers discovered that myeloid DC counts returned to normal by day 365 post‐HSCT, whereas plasmacytoid DCs continued to be present at lower frequencies than in the controls, which contributed to a higher incidence of developing infections [113]. Goncalves et al. [114] designed a multicenter prospective study to demonstrate that low DC counts, particularly plasmacytoid DC counts, following engraftment are significantly associated with a poorer prognosis. Notably, Chang et al. [24] reported that the number of DCs (including myeloid DC1s, myeloid DC2s and plasmacytoid DCs) in patients who underwent haplo‐HSCT with the Beijing protocol was significantly lower than that in HLA‐matched recipients on both day 15 and day 30 after transplantation because of the impact of ATG on DCs. For haplo‐HSCT with the PTCy protocol, the recovery of DCs has not been fully assessed.

#### 3.1.4 NK Cells

NK cells are an important type of innate immune system cell that represented the first reconstituted lymphocyte population 1–4 months after HSCT [5]. However, it takes longer for NK cells to function efficiently after HSCT [115]. NK cells have been shown to have important impacts on immune tolerance and surveillance, which are crucial for reducing the rates of relapse and infection [116]. The self‐recognition of NK cells involves inhibitory NK cell receptors such as killer immunoglobulin (Ig)‐like receptors (KIRs) and C‐type lectins such as CD94/NKG2A, which can bind specific alleles of MHC‐I. NK cells initiate an immune response to kill by activating NK cell receptors when MHC‐I is absent or underexpressed while preserving immune tolerance to tissues that express MHC [117]. NK cells can be characterized by the expression of CD56 and CD16 in CD3^−^ cells [118]. The most prominent subtype of NK cells is CD56^bright^CD16^dim^ NK cells, which represent NKG2A^−^KIR^+^ cells and are able to kill leukemia cells after transplantation. In haplo‐HSCT, mismatches between inhibitory NK cell receptors and HLA alleles on recipient cells cause alloreactivity after graft infusion into recipients. This alloreactivity allows donor NK cells to eliminate surviving recipient immune cells, which could help prevent graft rejection. Alloreactivity can also help NK cells to kill recipient APCs to avoid GVHD and clear residual malignant cells, which is referred to as the graft‐versus‐leukemia (GVL) effect [119].

Russo et al. [120] explored the reconstitution of NK cells in patients who underwent haplo‐HSCT with PTCy. After Cy infusion, a significant reduction in the number of proliferating NK cells was observed, indicating the selective elimination of dividing cells. Following the depletion of mature NK cells, immature NK cells become prevalent from day 15 post‐HSCT, likely originating from infused hematopoietic stem cells and supported by high interleukin‐15 (IL‐15) levels in patients' sera. Notably, the number of single KIR NK cells, potentially alloreactive, was also reduced by PTCy, leading to decreased numbers and antileukemic potential by day 30 post‐HSCT. Thus, the absolute counts and relative proportions of mature NK cells on day 30 post‐HSCT may more reliably predict effective NK cell‐mediated immunosurveillance against relapse after haplo‐HSCT with PTCy [120]. Interestingly, a study of NK cells and haplo‐HSCT with the PTCy protocol by Roberto et al. [115] revealed the defective cytotoxic function of a donor‐derived unconventional subset of NKp46^neg‐low^/CD56^dim^/CD16^neg^ NK cells because these cells express remarkably high levels of CD94/NKG2A.

Retiere et al. [97] reported fast NK cell reconstitution on day 30 after HSCT in both the ATG and PTCy groups, and a greater absolute count of NK cells was observed in the ATG group. However, no obvious difference in the main clinical outcomes, including OS, DFS, and relapse incidence, were observed between the two groups [97]. Later, Massoud et al. [31] compared the kinetics of NK cell recovery between a PTCy group and an anti‐T‐lymphocyte globulin (ATLG) group and reported that in the ATLG group, NK‐cell reconstitution was significantly faster by day 30, but by days 100 and 180, the levels were comparable between the ATLG and PTCy groups. Univariate analysis revealed that PTCy was associated with a greater incidence of NRM than was ATG in this study. The selective depletion of KIR‐alloreactive NK cells by Cy may partly account for the absence of favorable outcomes.

In summary, NK cell recovery analysis could be used to predict the clinical outcomes of patients who undergo HSCT, and increasing the number of NK cells could improve clinical outcomes by decreasing the incidence rates of relapse and infection [116]. Currently, NK cell functions are being harnessed to develop adoptive NK cell transfer therapies for solid and hematological tumors.

#### 3.1.5 γδT Cells

γδT cells make up approximately 1–10% of the total T‐cell population and usually reside in the mucosa and skin [121]. There are two main subsets of γδ T cells in human PB: the Vδ2 subset, which constitutes 50–90% of γδ T cells, and the Vδ1 subset, which is more common in the mucosal epithelium, such as the skin and intestine. Additionally, a minor Vγ3 subset exists in the PB, representing less than 0.1% of CD3^+^ T cells [122]. The multifaceted roles of γδT cells include both innate and adaptive immune functions, which play crucial roles in controlling inflammation and maintaining tolerance to self‐antigens, contributing to immediate tumor defense and the selective recognition of viral antigens and bacterial metabolites [123]. Moreover, these cells promote DC maturation and antibody production via B‐cell interactions. Consequently, γδT cells can play roles in both innate and adaptive immunity. During the initial weeks after transplantation, γδ T cells constitute the main T‐cell population; these γδ T cells are derived mainly from mature T cells derived from graft cells and expand in vivo within 2 weeks to 2 months [47, 124, 125]. With respect to haplo‐HSCT with the Beijing protocol, our group reported that the early‐stage recovery of Vδ2 T cells was consistently delayed after unmanipulated haplo‐HSCT [104, 126]. Massoud et al. [31] reported that the recovery of γδ T cells tended to be faster in the ATG group than in the PTCy group, which could partly explain the greater incidence of infection before day 100 in the PTCy group. Similarly, the number of γδ T cells was evaluated by Retiere et al. [97] in the ATG and the PTCy groups on day +60 post‐HSCT. Between days 0 and 30 after transplantation, the percentage of γδ T cells was significantly greater in the ATG group.

### 3.2 The Reconstitution of Adaptive Immune Cells

Unlike innate immunity, the reconstitution of adaptive immune cells is typically slower, with B‐cell and CD8^+^ T‐cell numbers normalizing between 100 days and 6 months post‐HSCT and thymic‐dependent CD4^+^ T‐cell reconstitution occurring between 6 and 9 months post‐HSCT (Figure 3). Initial T‐cell recovery involves peripheral expansion of CD8^+^ memory T cells from the donor graft or residual recipient T cells postconditioning. These expanded CD8^+^ T cells respond to cytokines and previously encountered viruses but have a limited capacity to respond to new antigens. Full T‐cell reconstitution depends on lymphoid progenitors differentiating in the thymus into naive CD4^+^ or CD8^+^ T cells with MHC‐restricted, antigen‐specific receptors [5, 110, 127, 128]. The kinetics of immune system reconstitution are correlated with posttransplantation morbidity, including infections, graft loss, and GVHD [8].

#### 3.2.1 T Cells

T cells, which rely on their T‐cell receptor (TCR) to recognize antigens presented by MHC, constitute a crucial subset of the adaptive immune system. The reconstitution of T cells depends on the output of thymic and homeostatic peripheral expansion (HPE). Since thymic function is disrupted by conditioning regimens, in the first 6 months posttransplantation, the recovery of T cells predominantly relies on HPE [35]. The dynamics of T‐cell reconstitution depend on patient and donor characteristics, as well as transplant factors. In a sequential multiomic analysis of skin and blood T cells during HSCT, Ram et al. [30] reported that epigenetic regulation plays a significant role in immune recovery, particularly in modulating T‐cell recovery across different tissues, such as blood and skin.

##### 3.2.1.1 CD4 ***^+^*** T cells

The reconstitution of CD4^+^ T cells is markedly slow after HSCT, with this T‐cell subset usually being the last to be reconstituted [15, 24, 25, 27]. In a study of the Beijing protocol, Chang et al. [24] reported that haploidentical recipients had lower T‐cell counts, especially CD4^+^ T‐cell counts, before day 90 posttransplantation than did HLA‐matched recipients. The median CD4^+^ T‐cell count remained below 200 cells/µL for the first 90 days post‐HSCT and increased to 200 cells/µL by day 180. The authors attributed the prolonged CD4^+^ T‐cell lymphopenia after haploidentical transplantation to ATG use, which could impair thymic function in these recipients. However, the CD4^+^ T‐cell counts at days 30 and 90 were not associated with survival in this study. This lack of impact on survival was attributed to compensatory monocyte and CD8^+^ T‐cell expansion, exacerbated GVL effects due to HLA mismatch, and better management of cytomegalovirus (CMV) reactivation and GVHD [24]. In a case series of haplo‐HSCT with PTCy, Raiola et al. [102] found that the recovery of CD4^+^ T cells was found to be relatively fast: the median CD4^+^ T‐cell counts on days +40 and +100 after BM transplantation (BMT) were 64 and 149 cells/µL, respectively.

In a study conducted by Retiere et al. [97], the percentage of CD4^+^ T cells was significantly greater in the PTCy group than in the ATG group, suggesting that cyclophosphamide targeted proliferating cells but maintained CD4^+^ T cells. Massoud et al. [31] reported a similar trend toward faster CD4^+^ T‐cell reconstitution in a PTCy group than in an ATLG group.

##### 3.2.1.2 CD8^+^ T Cells

The recovery of CD8^+^ T cells has been proven to occur earlier than that of CD4^+^ T cells [24, 25, 27]. Reconstitution of the T‐cell compartment after allo‐HSCT involves HPE of donor T cells and the production of new naive T cells in the thymus. HPE favors CD8^+^ T cells, which proliferate more effectively than CD4^+^ T cells do [32]. Chang et al. [24] reported that the median CD8^+^ T‐cell counts at 30, 90, 180, and 1 year after haplo‐transplantation with the Beijing protocol were 98, 672, 918, and 884 cells/µL, respectively. For haplo‐HSCT with PTCy, Raiola et al. [102] reported that the recovery of CD4^+^ T cells was relatively rapid: the median counts were 64/cmm on day 40 and 149/cmm on day 100 post‐HSCT, which were comparable to those of recipients from HLA‐identical siblings. Servais et al. [129] examined the impact of ATG on IR after allo‐HSCT and reported that ATG depleted naive CD4^+^ and CD8^+^ T cells without significantly affecting memory T cells. Massoud et al. [31] reported that CD8^+^ T cells were reconstituted faster than CD4^+^ T cells were, with CD8^+^ T‐cell recovery occurring by day 100. CD4^+^ T‐cell reconstitution was not observed by day 180. PTCy affected fewer CD4^+^ T cells but had greater activity against CD8^+^ T cells, leading to greater CD4^+^ T‐cell proliferation in the PTCy group and more CD8^+^ T cells in the ATLG group. Additionally, ATLG seemed to impact memory CD8^+^ and CD4^+^ T cells more than PTCy did [31].

##### 3.2.1.3 Treg Cells

Treg cells constitute a subgroup of CD4^+^ T lymphocytes that play crucial roles in dampening immune reactions and preserving self‐tolerance [130]. The transcription factor FoxP3 is essential for both Treg differentiation and functionality, serving as a definitive marker for their identification [131]. These cells are a fully developed subset of T lymphocytes and can also arise from CD4^+^CD45RA^+^ naive T cells in peripheral tissues [132]. Treg cells could suppress the functions of conventional CD4^+^ and CD8^+^ T cells, B cells, NK cells, and APCs, therefore they are believed to be crucial mediators in promoting immune tolerance and preventing GVHD both in mouse models and in human patients [133, 134, 135]. In patients who underwent haplo‐HSCT with PTCy, the CD4^+^Foxp3^+^ Treg cells of those patients rapidly reconstituted after transplantation through the expression of ALDH, which could resist the toxicity of Cy [94]. Moreover, Kanakry et al. [94] reported that mice treated with PTCy after Treg cell removal experienced weight loss, higher GVHD severity scores, and shorter survival. These findings indicate that Treg cells play a crucial role in the mechanism by which PTCy prevents GVHD. Similarly, a study conducted by Retiere et al. [97] revealed that the proportion of Treg cells was significantly greater in a PTCy group than in an ATG group. Surprisingly, Di Ianni et al. [38] proved for the first time in humans that the adoptive transfer of Treg cells can prevent GVHD, promote lymphocyte reconstitution, and enhance immunity against opportunistic pathogens without the need for posttransplant immunosuppression, while preserving the GVL effect.

#### 3.2.2 B Cells

In adaptive immunity, B cells are crucial for many immune responses. CD19^+^ pre‐B cells become pro‐B cells following Ig heavy chain rearrangement and then differentiate into immature B cells after light chain rearrangement [136]. After transplantation, B cells are not detected for up to +1 month, and the proportion of B cells reaches normal levels at +3 months, while it takes 6–12 months for the absolute count to reach normal levels [109, 110]. However, functional reconstitution may take longer, such as months to years [137]. During the initial months after transplantation, regenerating B cells exhibit functional incompetence, as evidenced by their lack of proliferative and differentiative responses to antigen‐specific factors [15].

Roberto et al. [109] revealed the maturation process of B cells after haplo‐HSCT with PTCy and identified four transitional subsets: T0 stage (CD5^−^ CD21^−^), T1 (CD5^+^ CD21^−^), T2 (CD5^+^ CD21^+^), and CD5^−^ CD21^+^. Five weeks after HSCT, most of the recovered B cells were T0 or T1 cells. Over time, the number of T0 cells decreased, whereas the number of T2 and CD5^−^ CD21^+^ cells increased. B‐cell maturation varied among donors and patients up to 14 weeks, with transitional B cells progressively expressing the naive markers IgD, IgM, and CD217. At week 15, IgM expression was significantly greater than that in donor transitional B cells [109]. Several researches have revealed that GVHD and CMV infection could delay the B cell recovery, as well as the treatment of glucocorticoids [138, 139, 140].

Some studies have reported that the application of ATG could probably delay the reconstitution of B cells. According to research conducted by Bosch et al.^15^, counts of total, naive, and memory B cells were generally lower after ATG‐based conditioning than after non‐ATG‐based conditioning in the first month posttransplantation. The difference was significant for all subsets except for memory B cells [15, 138]. However, some researchers reported that the use of ATG, as well as the HLA disparity, did not have influence on the reconstitution of B cells [24, 138]. Massoud et al. [31] reported that the reconstitution of B cells was comparable in both the ATG and the PTCy groups; however, there was a trend toward faster reconstitution of naive B cells (CD19^+^CD27^−^CD10^+^) in the PTCy group [31].

Several studies have suggested that low B‐cell counts are correlated with higher infection rates and rates of major complications, highlighting the importance of early and effective B‐cell reconstitution [36, 138, 141]. Further studies using clinical samples and animal models would increase our understanding of the etiology of abnormal B‐cell homeostasis and aid in the development of therapeutic and prophylactic approaches for complications after transplantation.

Moreover, immune cell subsets do not function in isolation; rather, they engage in complex interactions. We believe that the interplay among T cells, B cells, DCs, NK cells, monocytes‐macrophages, and γδ T cells collectively shapes the dynamic process of IR. For example, accumulating evidence has demonstrated that naive B cells can drive the differentiation of naive CD4⁺ T cells into CD25⁺Foxp3⁻ Treg cells [142]. Moreover, B cells play a critical role in maintaining Treg homeostasis and collaborate with Treg cells to ameliorate inflammation. Together, these findings highlight the integral role of B cells in modulating T‐cell‐mediated immune regulation [143], which could have significant implications for the development of novel therapeutic strategies to control inflammation. At present, relatively few studies have specifically examined the interactions among cell subsets following transplantation. Future research into these intercellular interactions will help optimize immunomodulatory strategies, improve transplant success rates, and reduce complications.

## 4 The Role of IR in Clinical Outcomes

HSCT serves as a critical treatment option for hematological disorders. However, posttransplant immune deficiency remains a leading contributor to morbidity and mortality. Previous findings indicate that during the initial phase after transplantation, neutropenia significantly elevates susceptibility to opportunistic infections. The recovery of B cells, and particularly T cells, is notably sluggish, leading to prolonged immunodeficiency that may persist for 1–2 years posttransplantation. This delayed IR has been linked to multiple complications, including increased infection risk, increased likelihood of malignant relapse, and challenges such as mixed chimerism and graft rejection due to the persistence of host T cells. We summarize the cellular subsets that exhibit are closely associated with major posttransplant complications, aiming to facilitate posttransplant monitoring and early prediction of complications (Table 2).

### 4.1 Relapse

Many studies have revealed correlations between the IR of certain immune cell subsets and disease relapse [144, 145]. In patients undergoing haplo‐HSCT, the GVL effects of NK cells and T cells are expected to increase due to HLA mismatches between donors and recipients. Turcott et al. [146] reported that a higher monocyte count on day +28 was associated with a lower incidence of relapse after allo‐HSCT. In a study conducted by Chang et al. [147], for patients who underwent haplo‐HSCT with the Beijing protocol, an absolute lymphocyte count (ALC) of more than 300 cells/µL on day 30 post‐HSCT was associated with lower rates of relapse. Similarly, in a study of 66 patients undergoing haplo‐HSCT with the PTCy protocol, Perez‐Corral and colleagues [148] reported that the ALC on day 30 was an independent prognostic factor in terms of relapse‐free survival.

As the earliest lymphocyte subset to reconstitute following HSCT, NK cells are crucial for preventing early relapse [149]. Russo et al. [120] conducted a comprehensive study of NK cell reconstitution in patients who underwent haplo‐HSCT with the PTCy protocol. Researchers reported that single KIR^+^ NK cells, which may include potentially alloreactive NK cells, were also depleted by PTCy, leading to reduced antileukemic activity by day 30 post‐HSCT. A larger analysis of 99 patients who underwent haplo‐HSCT with PTCy revealed that the absolute count and proportion of mature NK cells on day 30 may serve as a more accurate predictor of effective NK cell‐mediated immunosurveillance against relapse after haplo‐HSCT with PTCy. Moreover, Chang et al. [101] reported a connection between lower rates of relapse and quicker quantitative recovery of CD56^bright^ NK cells. NK cells mediate their antileukemic effects through multiple pathways, including direct target cell lysis via perforin and granzyme B, as well as indirect mechanisms such as antibody‐dependent cellular cytotoxicity and the secretion of proinflammatory cytokines and chemokines. KIRs were found to be crucial components in the regulation of both self‐tolerance and antitumor responses. KIR ligand mismatches between grafts and hosts lead to “missing self” recognition by donor NK cells, reducing relapse risk and improving engraftment and GVHD protection in patients with AML [150]. In children with acute leukemia, NK cell alloreactivity from haploidentical donors was also found to lower the risk of relapse [151].

The beneficial impact of elevated CD8^+^ T‐cell counts on mitigating disease relapse risk has been well documented in retrospective studies, which revealed that a CD8^+^ T‐cell count exceeding 50 × 10^6^ cells/L on day 28 after HLA‐matched transplantation was correlated with a reduced risk of relapse. Conversely, in a haploidentical setting, Tian et al. [28] reported no direct correlation between CD8^+^ T‐cell counts and relapse rates. However, their study revealed that patients who achieved CD8^+^ T‐cell counts of 375 × 10^6^ cells/L by day 90 post‐HSCT experienced reduced NRM and longer leukemia‐free survival [28].

### 4.2 Infections

Improving and accelerating immunological recovery is crucial for preventing infections in recipients after HSCT [152]. Some studies have shown that the distinct reconstitution characteristics of immune cell subsets from different conditioning regimens are associated with varying infection profiles. Viral infections, especially CMV infection and Epstein‒Barr virus (EBV) infection, are the most common type of opportunistic infection after transplantation and are a significant cause of multiorgan dysfunction [153].

It is reported that there was a close association between virus infection and impaired T cell reconstitution. In studies of haplo‐HSCT with the Beijing protocol, our group found that the early‐stage recovery of Vδ2 T cells was consistently delayed after unmanipulated haplo‐HSCT, which was significantly associated with EBV reactivation [104, 126]. A study by Huang et al. [154] demonstrated that the proportions of CD8⁺ and CD4⁺CD45RO⁺ T cells at 30 days post‐HSCT were associated with the risk of EBV reactivation. Similarly, Lin's [155] results showed that at 1‐month posttransplant, the proportion of CD3⁺CD8⁺ T cells was lower in EBV⁺ patients than in EBV⁻ patients. Chang et al. [24] evaluated a group of patients in whom the Beijing protocol was used and reported a higher CMV antigenemia rate (49.9%) following haplo‐HSCT than following HLA‐matched HSCT (13%), with delayed reconstitution of DCs and T cells. However, the incidence of CMV disease was lower after haplo‐HSCT, likely due to the expansion of CMV‐specific central memory CD45RO^+^ CD62L^−^ CD8^+^ T cells, which mature into effector memory T cells in response to CMV antigenemia. According to a meta‐analysis, high γδT cells post‐HSCT are associated with a lower incidence of infection [106]. Moreover, γδT cells have been suggested to be directly involved in the response to anti‐CMV immune conditions [125]. Recently, the use of new approaches, such as CMV‐specific cytotoxic T lymphocytes, could help rebuild effective reconstitution of the cellular immunity against CMV [156]. A comparative study conducted by Massoud et al. [31] revealed that a PTCy arm experienced a greater incidence of infections before day +100, whereas an ATG arm exhibited greater reactivation of EBV. The reactivation rates of CMV were similar between the groups [31]. The authors attributed the higher rates of total infections to the slower recovery of NK cells and NKT cells in the PTCy group.

For bacterial and fungal infections, a similar connection was also observed between increased infection and weak T cell recovery. However, among patients with CD8^+^ T‐cell counts less than 375 cells/µL on day 90, the bacterial infection rate was higher at 41.6%, whereas it was 14.6% in those with CD8^+^ T‐cell counts above 375 cells/µL [28]. Moreover, Hong et al. [100] reported that impaired neutrophil maturation reduced macrophage inflammation modulation, contributing to sepsis pathogenesis. Infectious mortality rates ranged from 9 to 12% after haplo‐HSCT with the PTCy protocol, accounting for the majority of NRM events post‐HSCT. Bacterial infections were reported in 62–63% of patients and were associated with increased NRM [157]. Fungal infections were less prevalent, affecting 10–12% of patients primarily during the initial hospitalization, with only one case occurring 1 year after HSCT.

Moreover, although B cells recovered more slowly than T cell, B cells still played important protective roles in infection defense. A deficiency in B‐cell recovery was associated with infection within a short or long period after transplantation, ranging from 80 days or 7 years [158, 159, 160].

### 4.3 Graft‐Versus‐Host Disease

Treg cells prevent GVHD by suppressing the expansion and activation of alloreactive T cells in the proinflammatory environment induced by conditioning regimens [161]. They inhibit CD4^+^ and CD8^+^ T‐cell proliferation, downregulate IL‐2Rα expression, and reduce the levels of inflammatory cytokines, such as IFN‐γ and TNF‐α [162]. This suppression limits donor T‐cell expansion in GVHD target tissues while preserving GVL effects, ensuring tumor elimination without impairing IR. These mechanisms support the clinical use of Treg cells to improve transplantation outcomes, particularly in mismatched donor settings [163]. Studies have shown that higher counts of Treg cells in the PB of patients post‐HSCT are correlated with a lower incidence of aGVHD when standard prophylactic regimens are used [164, 165]; however, these results were controversial, which was likely due to the different protocols used.

In addition, early clinical trials have shown that Treg cell administration could modulate GVHD in patients after transplantation [152, 166, 167]. Trzonkowski et al. [168] first applied this treatment in human and demonstrated that adoptive transfer of ex vivo expanded CD4⁺CD25⁺CD127⁻ Treg cells alleviated symptoms and reduced immunosuppression in chronic GVHD. Recently, more studies have shown that the adoptive transfer of Treg cells after haplo‐HSCT could prevent GVHD without any posttransplantation immunosuppression, and without altering the GVL effect [38, 169]. A phase II randomized clinical trial conducted by Gao et al. [170] revealed that the infusion of mesenchymal stromal cells may decrease the incidence of chronic GVHD by increasing the number of Treg cells. Similarly, a study including 42 patients who underwent haplo‐HSCT draw a conclusion that Galectin‐9 may ameliorate aGVHD by modulating the Treg/effector T‐cell balance and regulating the PI3K/AKT/mTOR pathway [171]. Recently, sirolimus, an mTOR inhibitor, has been clinically applied to promote the in vivo expansion of Treg cells by converting CD4⁺CD25⁻ naive T cells into CD4⁺Foxp3⁺ Treg cells, thereby controlling the activity of effector T lymphocytes that cause GVHD [172].

Although rapid recovery of T and NK cells can be beneficial for tumor control and infection prevention, an accelerated increase in the number of T cells may increase the risk for aGVHD posttransplantation. For patients undergoing haplo‐HSCT with the Beijing protocol, Chang et al. [101] reported that a high T/NK ratio (>1.0) was an independent predictor of aGVHD and cGVHD. This finding aligns with that of Soiffer, who reported that patients with elevated CD8^+^ T‐cell counts or reduced CD56^+^ NK‐cell counts during the second week after BMT faced a greater risk of aGVHD [173]. Chronic GVHD may involve autoreactive T cells that evade thymic selection and are influenced by preconditioning or aGVHD. NK cells can suppress CD8^+^ T‐cell‐mediated autoantibody production through TGF‐β. Thus, a low NK‐cell count relative to that of T cells (T/NK > 1.0) may be insufficient to control autoantibody synthesis.

Iwamoto et al. [108] analyzed B cells at 8 weeks after transplantation and reported that haplo‐HSCT with PTCy was associated with a high proportion of transitional B cells and a low proportion of switched‐memory subsets. Conversely, haplo‐HSCT with ATG was associated with a high proportion of switched‐memory B cells and a low proportion of transitional subsets. Analysis of the incidence of cGVHD revealed that a greater percentage of transitional B cells was significantly associated with a lower risk of cGVHD. The impaired reconstitution of naive B cells was associated with the development of cGVHD [108]. These findings indicate that PTCy can reestablish favorable B‐cell homeostasis by promoting naive B‐cell emergence from the BM and suppressing early B‐cell activation in peripheral lymph nodes. Taken together, these findings suggest that early abnormal B‐cell lymphopoiesis is a key factor in cGVHD development, making the PTCy protocol a promising strategy for preventing this abnormality and achieving long‐term immune tolerance because this protocol is believed to create an immunologically calm environment by efficiently depleting alloaggressive effector T cells while sparing Treg cells [108].

## 5 Strategies to Improve Immune Cell Reconstitution after haplo‐HSCT

It could be concluded that IR is tightly linked to reduced complication risks and increased OS following haplo‐HSCT. Therefore, the development of treatment strategies to better predict and improve IR is crucial for optimizing patient outcomes. To further optimize immune cell reconstitution, researchers have explored the application of cytokine therapies and adoptive immunotherapies [34, 174] (Table 3). For example, therapy with cytokines, such as IL‐2, IL‐7, IL‐15 and growth factors, including keratinocyte growth factor (KGF), can promote immune recovery by regulating T‐cell proliferation and differentiation of T cells. Moreover, adoptive immunotherapy introduces specific immune cells, such as NK cells and Treg cells, to help modulate immune responses and reduce posttransplantation relapse rates. The application of these innovative therapeutic approaches paves the way for personalized treatments, contributing to better immune recovery and patient outcomes after transplantation.

**TABLE 3:** Strategies to enhance immune reconstitution after HSCT.

Therapies	Target cells	Benefits	Preclinical research	Relative clinical trial(s)
Cytokines				
IL‐2	NK cells; Treg cells	Ameliorates GVHD without exacerbating the GVL effect; reduces relapse	Thiolat et al. [175]	NCT03862833; NCT00539695; NCT01517347; NCT00529035
IL‐7	HSPCs; Thymocytes; Peripheral T cells	Remodels of immune homeostasis; minimizing GVHD; enhances TCR diversity.	Alpdogan et al. [176]	NCT30941769; NCT00684008
IL‐15	NK cells; CD8^+^T cells; B cells	Promotes GVL without GVHD effects	Chen et al. [177]	/
Growth factors				
KGF	TECs	Facilitates peripheral T‐cell reconstitution	Min et al. [178]; Ruth et al. [179]	NCT00031148; NCT00041665
IGF‐1	TECs	Enhances lymphoid and myeloid reconstitution after HSCT without aggravating GVHD	Alpdogan et al. [180];	/
rhGH	TECs	Leads to an overall increase in thymocytes; enhances thymic function and peripheral immune responses	Ahuva et al. [181]	NCT00737113
Cell‐based approaches				
NK cells	/	Enhances the GVL effect without aggravating GVHD	Lundquvist et al. [182]; Ruggeri et al. [150]	NCT06641648; NCT00303667; NCT02477787
Treg cells	/	Maintains immune tolerance and prevents GVHD	Nguyen et al. [183]	NCT04678401; NCT01927120
MSC	Bone marrow stromal cells; NK cells	Enhances hematopoietic engraftment; decreases incidences of GVHD	Muriel et al. [184]	ChiCTR‐IIR‐16007806; ChiCTR‐IOR‐15006330
Virus‐specific T cells	Specific virus	Clears virus infection	Pei et al. [185]; Reddehase MJ et al. [186]	NCT03378102; NCT02982902; NCT04056533; NCT02227641

Abbreviations: CAR‐T: chimeric antigen receptor T‐cell; DLI: donor lymphocyte infusion; GVHD: graft‐versus‐host disease; GVL: graft‐versus‐leukemia; HSCT, hematopoietic stem cell transplantation; HSPCs: hematopoietic stem and progenitor cells; IGF1, insulin‐like growth factor 1; IL, interleukin; KGF, keratinocyte growth factor; NK, natural killer; rhGH: recombinant human growth hormone; TCR: T‐cell receptor; TEC, thymic epithelial cell.

### 5.1 Exogenous Cytokine and Growth Factor Therapy

#### 5.1.1 Interleukin 2

Administering recombinant IL‐2 as a consolidative immunotherapy early after HSCT at a time of minimal residual disease (MRD), may enhance immunocompetence and reduce relapse rates in these patients. This effect is primarily due to the ability of IL‐2 to drive T‐cell differentiation into effector cells while promoting the proliferation of NK cells. Exogenous low‐dose IL‐2 can modulate immunity by increasing the number of circulating Treg cells, ameliorating GVHD without exacerbating the GVL effect, as shown in several animal experiments [175, 187, 188]. Moreover, in a recent clinical trial conducted by Kennedy‐Nasser et al. [189], the effect of ultralow‐dose IL‐2 on GVHD was evaluated. The authors reported that Treg levels significantly increased following therapy, especially in MRD transplant recipients. None of the patients treated with IL‐2 developed Grade II–IV aGVHD, and the number of viral infections was significantly lower in the IL‐2 group than in the comparator group [189]. Interestingly, Thiolat et al.[175] reported a significant reduction in exhausted CD8^+^ T cells in mice treated with pro‐Treg IL‐2Cx, indicating a strong antitumor effect. Currently, the standard protocol and dosage for IL‐2 administration still require further exploration and evaluation. Several ongoing or completed clinical trials, such as “Zoledronic Acid in Combination with Interleukin‐2 to Expand Vγ9Vδ2 T Cells After T‐replete Haplo‐identical Allotransplant” (http://ClinicalTrials.gov, NCT03862833), have applied IL‐2 in the haploidentical HSCT setting to potentially decrease complications.

#### 5.1.2 Interleukin 7

IL‐7 is important for the development of T cells and facilitates thymic‐dependent pathways by repairing the damaged thymus [190, 191]. Studies have shown that reconstitution through the thymus‐dependent central T‐cell pathway plays a critical role in maintaining long‐term immune balance [190]. The success of this pathway can significantly reduce the risk of posttransplantation complications, particularly in minimizing GVHD. Some animal studies have shown that IL‐7 significantly promotes T‐cell IR after transplantation [190, 191, 192, 193], and that its effects increase with MHC incompatibility [194], indicating that IL‐7 administration may be beneficial for the remodeling of immune homeostasis after haploidentical transplantation. IL‐7 administration has been demonstrated to safely improve lymphoid reconstitution in recipients undergoing allo‐HSCT without exacerbating the risk of GVHD [190]. Currently, few clinical trials on the application of IL‐7 in HSCT are ongoing. A phase I trial conducted by Perales et al. [195] indicated that a short course of IL‐7 therapy could lead to an increase in the number of functional T cells, including CMV‐specific T cells. Enhanced TCR diversity was also observed after treatment. However, this trial was performed in patients after at allo‐HSCT with a TCD strategy platform.

#### 5.1.3 KGF and Insulin‐Like Growth Factor 1

KGF, a member of the fibroblast growth factor (FGF) family, plays a crucial role in promoting epithelial cell proliferation and differentiation by interacting with a splice variant of the FGF receptor (FGFR) expressed in various epithelial tissues, including the thymus [196]. Systemic administration of recombinant human KGF has been shown to provide significant cytoprotection to epithelial tissues in animal models of chemotherapy/radiation‐induced damage, particularly in the gastrointestinal tract, lungs, urinary bladder, and hair follicles. The protective effects of KGF on the thymus are likely mediated by increased intrathymic IL‐7 production by thymic epithelial cells. The potential of KGF to safeguard or improve thymic stroma regeneration in patients undergoing HSCT has been investigated in several studies [178]. Notably, KGF administration prior to both syngeneic and allogeneic HSCT has been shown to increase thymopoiesis and facilitate peripheral T‐cell reconstitution, highlighting the therapeutic potential of KGF administration to improve immune recovery posttransplantation [178, 197].

Moreover, insulin‐like growth factor 1 (IGF‐1) is one of the neuroendocrine factors with multiple functions, including promoting hematopoiesis, prolonging lymphocyte survival, and regulating T‐cell signaling. IGF‐1 has been demonstrated to enhance thymic regeneration in mice after HSCT, thereby facilitating peripheral immune cell recovery [180, 198].

### 5.2 Cellular Therapy

The adoptive transfer of ex vivo‐expanded immunomodulatory cell populations, including Treg cells, NK cells, and mesenchymal stem cells (MSCs), as well as antigen‐specific allogeneic T cells that target viral or tumor antigens, has demonstrated significant potential in improving IR following HSCT [199, 200, 201, 202]. These strategies offer promising avenues for improving posttransplantation immune recovery and reducing the incidence of complications such as infection, GVHD and relapse.

The infusion of unstimulated donor lymphocytes is an established cellular therapy that can induce long‐term remission and reduce relapse risk; however, this method also increases the risk of GVHD. Recently, some novel cellular therapies have shown tremendous potential. NK‐cell therapy is well tolerated when used as adoptive immunotherapy in patients undergoing haplo‐HSCT [203, 204, 205]. A pilot study by Passweg et al. [203] verified the feasibility of NK‐cell purification and infusion (NK‐DLI) in patients after haplo‐HSCT. Recently, Lee et al. [206] conducted a randomized clinical trial to evaluate the effect of donor‐derived NK cell infusion after haplo‐HSCT in patients at high risk of myeloid malignancy. Intention‐to‐treat analysis revealed reduced disease progression in the donor NK‐cell infusion group, with a 30‐month cumulative incidence of 35%, compared with 61% (*p* = 0.040; subdistribution HR, 0.50) [206].

Treg cells are critical mediators in the prevention of aGVHD and play essential roles in maintaining immune tolerance and suppressing alloreactive immune responses following transplantation [183, 207]. In a murine model of GVHD induced by conventional T cells, Nguyen et al. [183] reported that the adoptive transfer of Treg cells effectively mitigated the onset of GVHD, preserved the structural integrity of the thymus and peripheral lymph nodes, and accelerated donor lymphoid reconstitution, promoting a diverse TCR‐Vβ repertoire. Di Ianni et al. [38] demonstrated for the first time in humans that the adoptive transfer of Treg cells could prevent GVHD without any posttransplantation immunosuppression, promote lymphoid reconstitution, increase immunity to opportunistic pathogens, and maintain the GVL effect.

Viral reactivation remains a significant concern for transplant recipients, as refractory viral infections can be fatal, and antiviral drugs may cause renal failure and cytopenia [208]. A promising strategy for managing posttransplant viral reactivation is the infusion of donor‐derived virus‐specific T lymphocytes to restore antiviral immunity. In 1992, Riddell et al. [209] first demonstrated that CMV‐specific donor T‐cell clones could transfer immunity post‐SCT, successfully treating CMV reactivation. Following these proof‐of‐concept studies, key advancements led to clinically viable cellular therapies for controlling viral reactivation, primarily focusing on T‐cell therapies for CMV and EBV. A major breakthrough was the development of infusion‐ready donor‐derived cells lacking alloreactive responses without requiring pure CMV‐specific T‐cell clones. This can be efficiently achieved by selecting CMV pp65 peptide‐activated T cells via IFN‐γ capture or by targeting CMV antigen‐specific T cells bound to HLA class I multimers [210, 211]. Unfortunately, the need to generate patient‐specific T‐cell lines makes this approach impractical for widespread or urgent use. HLA partially matched related or unrelated donors can serve as third‐party donors, providing an alternative source of T cells with proven safety and clinical efficacy [212, 213]. Pei et al. [185] indicated that in murine models, both adoptively transferred donor‐derived and third‐party CMV‐specific cytotoxic T lymphocytes (CMV‐CTLs) migrate to virus‐infected or tumor‐infiltrated organs, persist for at least 28 days, and contribute to significant reductions in CMV pathology and viral load in target organs. Furthermore, her study also demonstrated clinical data from 31 patients receiving third‐party CMV‐CTLs and 62 matched patients receiving transplant donor‐derived CMV‐CTLs further support that these two CMV‐CTL strategies induce comparable antiviral responses [185].

## 6 Conclusion and Perspectives

This review provides a comprehensive overview of IR dynamics following haplo‐HSCT with non‐TCD protocols, focusing on the Beijing protocol and the PTCy strategies. In summary, accelerated IR has consistently been associated with better clinical outcomes in patients receiving haplo‐HSCT. This fact highlights the potential of monitoring immune cell remodeling as a valuable tool for guiding preemptive therapeutic interventions and enabling the timely administration of treatments to minimize the incidence of complications, thus improving patient prognosis.

Achieving and maintaining an optimal immune recovery is crucial for posttransplant success. It could help us to strike a balance between GVHD and GVL effect, as well as handle complications such as GVHD, infection, and relapse, which could lead to improved long‐term outcomes and life quality of patients receiving haplo‐HSCT. In the future, the use of new biomarkers and predictive tools to forecast immune recovery dynamics for each patient will be a fundamental component for clinicians to design personalized protocols of haplo‐HSCT.

In conclusion, continued exploration of IR patterns and mechanisms is essential—not only for identifying patients who may benefit from early immune interventions during HSCT, but also for developing effective immunotherapies to enhance and accelerate immune recovery in clinical practice.

## Author Contributions

Xiaojun Huang designed the review. Xiaodi Ma and Zhengli Xu wrote the original draft and revised the manuscript. All authors approved the final version for submission.

## Conflicts of Interest

The authors declare no conflicts of interest.

## Data Availability

The authors have nothing to report.
